# THE IMPACT OF WORKPLACE DEMANDS AND RESOURCES ON THE JOB SATISFACTION OF MINORITY AND IMMIGRANT CNAS

**DOI:** 10.1093/geroni/igad104.3646

**Published:** 2023-12-21

**Authors:** Frances Hawes, Shuangshuang Wang

**Affiliations:** University of Wisconsin-Eau Claire, Eau Claire, Wisconsin, United States; Southwest Jiaotong University, Chengdu, Jiangsu, China (People’s Republic)

## Abstract

This study examines the association between occupational stress (job resources and demands) and job satisfaction among nursing assistants (NAs) in the long-term care (LTC) sector, considering differences based on race/ethnicity and immigration status. Data from 2,763 NAs were retrieved from the National Nursing Assistant Survey (NNAS). Guided by the JD-R model, our findings indicate that higher job resources, including organizational support, personal development opportunities, benefits, and peer support, are associated with greater job satisfaction among NAs. Conversely, higher job demands, such as structural, physical, and emotional demands, are linked to lower job satisfaction. Importantly, significant differences in occupational stress were observed based on race/ethnicity and immigration status. Hispanic NAs reported the lowest benefits, Black NAs reported the lowest levels of residents’ respect, and White NAs reported the lowest peer support. Naturalized NAs experienced higher job satisfaction with greater job resources, while resident NAs had lower satisfaction associated with higher job demands. These findings highlight the need for tailored support mechanisms to address disparities in job rewards and recognition among different racial/ethnic and immigration groups.

